# Evaluating impact of possible transgenic poplar cultivation on protected areas

**DOI:** 10.1186/1753-6561-5-S7-P182

**Published:** 2011-09-13

**Authors:** Anna Buonamici, Donatella Paffetti, Davide Travaglini, Stefano Biricolti, Francesca Bottalico, Lorenzo Chelazzi, Filippo Cimò, Isabella Colombini, Silvia Fiorentini, Valeria Tomaselli, Cristina Vettori

**Affiliations:** 1 Plant Genetics Institute, CNR, UOS FI, Via Madonna del Piano 10, 50019 Sesto Fiorentino (FI), Italy; 2 Department of Agricultural and Forest Economics, Engineering, Sciences and Technologies, University of Florence, Via San Bonaventura 13, 50145 Florence, Italy; 3 Department of Agronomy and Land Management, University of Florence, piazzale delle Cascine 22, Florence, Italy; 4 Institute of Ecosystem Study – CNR, UOS FI, Via Madonna del Piano 10, 50019 Sesto Fiorentino (FI), Italy; 5 DG Competitiveness of regional system and development of competencies, Regional Government of Tuscany, Via di Novoli 26, 50127, Firenze, Italy; 6 Plant Genetics Institute, CNR, Via Amendola 165/A, 70126 Bari, Italy

## Background

Plant biodiversity studies have been performed in the Migliarino-San Rossore-Massaciuccoli Regional Park in Tuscany (Italy) within the framework of the European project LIFE08 NAT/IT/342.This project aims at developing a quick monitoring index (QMI) to rapidly assess the potential risk generated by transgenic plants in characterized ecosystems or biotopes. For this reason test areas have been selected inside the protected area to evaluate plant (weeds and trees), animal, and soil microoganisms biodiversity. The proximity of the selected test area to cropped surfaces where Genetically Modified Plants (GMPs) might be cultivated has been taken into account. GMPs could spread pollen and contaminate natural populations. To avoid this risk, an efficient monitoring system is required taking into account genetic diversity and breeding study. As far as tree biodiversity concern, *Populus* species were identified in the test areas. Two populations of *Populus* present into two different ecosystems (forest and wetland areas) were examined together with two cultivated varieties. The two ecosystems were characterized for the vegetation. Nuclear microsatellites were used to evaluate genetic diversity of poplar populations and level of breeding between natural and cultivated *Populus*. In addition the insect populations present on male and female poplars during flowering period have been studied.

## Materials and methods

The selected *Populus* test areas are: A) a mixed forest stand in the Tenuta di San Rossore; B) a scattered *Populus* population in the wetland area of “lake of Massaciuccoli”. Test areas A and B are 8 Km apart.

Test area A is a naturally-originated mixed forest stand. The prevailing tree species are *Populus alba*, *P.* x *canescens*, *Fraxinus angustifolia* and *Alnus glutinosa*. In the test area B single trees and small groups of *Populus* spp. are scattered along the lake.

In the test area A an experimental subplot 2500 m^2^ large has been designed and the position has been acquired by GPS. All *Populus* trees within the plot have been identified and their position have been collected by GPS. The stand structure analysis has been performed using spatial functions.

In the test area B the position of *Populus* has been acquired by GPS.

The poplar populations present in areas were examined. From literature, six nuclear microsatellites were selected, all markers carried dinucleotides repeats except two with trinucleotides repeats [1,2].

Total DNA was extracted from the samples to perform microsatellite analysis [1,2]. Sizing of the PCR products was carried out using software Gene Mapper ver. 4.0 (Applied Biosystem).

In order to better define the species density and composition of the herbaceous stratum, some sampling subplots have been designed within the experimental plot by using the standardized multi-scale approach proposed by Dengler [3]. To define possible variations of distribution and/or density of plant species, their relative abundance has been evaluated according to the Braun Blanquet approach (1964).

During poplar tree blooming period inflorescences of male and female trees were collected. Weekly sampling was carried out with the aid of an elevator truck that permitted to collect samples out of reach.

## Results

In relation to the herbaceous stratum, the observations made so far show some variability in the distribution of species to a plot to another, probably due to the micro topological characteristics of the site.

The biodiversity analysis of the insects present on poplar trees indicates an evident difference in the faunal community between male and female trees and a relatively low number of species. Dipteran larvae were most abundant followed by coleopterans such as coccinellids and curculionids, araneids and lepidopteran larvae.

The analysis of spatial and genetic structure of the two poplar populations was performed using Geneland [4].

**Figure 1:**
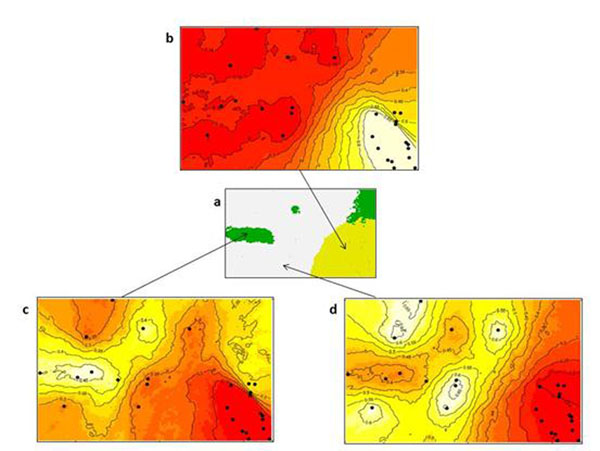
Results of Geneland analyses of area A showing: (a) Spatial organisation into three clusters; (b), (c), and (d) are maps of posterior probabilities of each cluster.

**Figure 2:**
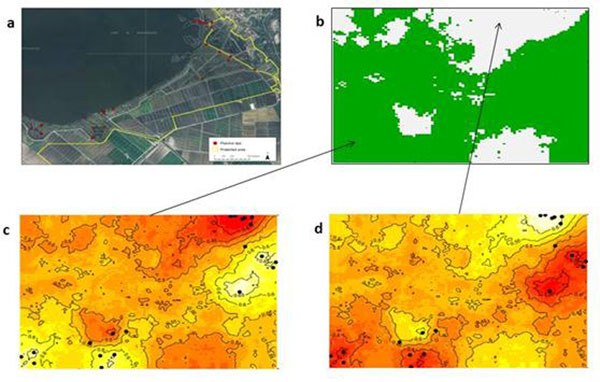
Map of area B (a), and results of Geneland analyses of area B showing: (b) Spatial organisation into two clusters; (c), and (d) are maps of posterior probabilities of each cluster.

The results of Geneland clearly showed that three distinct clusters can be identified in the area A (Figure 1a) indicating the presence gene flow barriers (Figures 1b, 1c, 1d). The cluster represented in Figure 1b comprise individuals with the same genotype as indicated by microsatellites data (not shown). In fact, it is highly probable that these individuals are root suckers. The second and third clusters show the occurrence of gene flow within and among the two clusters (Figures 1c and 1d). This indicates that a minor barrier is present because the other tree species of the stand block only in part the pollen flow. The same analysis indicates two clusters in area B (Figure 2) with a gene flow among them higher respect to the area A. In fact, this population is in an open area (lake) and therefore the pollen flow is favored.

## Conclusions

In general the level of biodiversity within the selected test area is high, and in particular the level of gene flow inside area B. Therefore, the possible cultivation of transgenic poplar close to the protected areas could influence their biodiversity. Especially the level of gene flow can determine a contamination of the autochthon poplar populations. The development of a QMI using the experimental data is in course.

The availability of the QMI to rapidly assess the potential risk generated by transgenic plants in characterized ecosystems, is a useful tool to assess the releasing or not of the permission of cultivation close to protected areas.

## References

[B1] Tuskan GA Gunter LE Yang ZK Tongming Y Sewell MM Difazio SP Characterization of microsatellites revealed by genomic sequencing of *Populus trichocarpa* Canadian Journal of Forest Research 2004 34 1 85 93 10.1139/x03-283

[B2] Smulders MJM Van Der Schoot J Arens P Vosman B Trinucleotide repeat microsatellite markers for black poplar (*Populus nigra* L.) Molec. Ecol. Notes 2001 1 3 188 190

[B3] Dengler J A flexible multi-scale approach for standardised recording of plant species richness patterns 2009 9 Ecological Indicators 1169 1178

[B4] Guillot G Estoup A Mortier F Cosson JF A Spatial Statistical Model for Landscape Genetics Genetics 2005 170 1261 1280 10.1534/genetics.104.033803 15520263 PMC1451194

